# Is the VITAMINS RCT indicating potential redundancy between corticosteroids and vitamin C?

**DOI:** 10.1186/s13054-020-02853-2

**Published:** 2020-04-06

**Authors:** Anitra C. Carr

**Affiliations:** Department of Pathology & Biomedical Science, University of Otago, Christchurch, PO Box 4345, Christchurch, 8140 New Zealand

Since the publication of the retrospective before and after study by Dr. Marik and colleagues, in which administration of hydrocortisone, intravenous (IV) vitamin C, and thiamine was indicated to provide a survival advantage in septic patients [1], there has been a prevalence of clinical trials of variable quality and subsequent meta-analyses indicating variable outcomes [2]. Unlike previous combination trials, which did not control for corticosteroid use in the control arm, the recently published VITAMINS randomized clinical trial standardized corticosteroid administration in the control arm to determine if IV vitamin C and thiamine could provide an additional survival advantage over hydrocortisone alone [3]. This trial showed no benefit of the combination of hydrocortisone, IV vitamin C, and thiamine in comparison to hydrocortisone alone.

Although the accompanying editorial by Dr. Kalil suggested that in light of this finding, IV vitamin C has no place in critical care, limitations of the VITAMINS trial preclude this view. It is well established that septic patients exhibit a high prevalence of vitamin C deficiency, and a recently published clinical trial in JAMA indicated that IV vitamin C may provide a survival advantage in these patients [4]. Since the VITAMINS trial did not include an arm with IV vitamin C monotherapy, this trial does not provide any information as to whether IV vitamin C is of benefit to septic patients in the absence of corticosteroid administration. This trial simply indicated that short-term IV vitamin C administration (mean of 3.4 days) may not be of additional benefit if corticosteroids are being administered to the patients. However, routine corticosteroid administration to septic patients is not a common clinical practice in all intensive care units.

Indeed, the VITAMINS trial may in fact indicate redundancy between vitamin C and corticosteroids. Vitamin C is thought to play a role in the stress response as evidenced by its very high concentrations in the adrenal glands and its release in response to ACTH [5]. Furthermore, animals that cannot synthesize vitamin C have significantly elevated cortisol release in response to stress compared with animals that can synthesize vitamin C endogenously (reviewed in [5]). Thus, administration of corticosteroids to septic patients may help compensate for deficient vitamin C concentrations in these patients. Alternatively, in the absence of corticosteroid administration, supplementation of deficient patients with vitamin C may help with their stress response and could potentially provide a survival advantage, particularly in patients with HPA axis dysregulation.

## Data Availability

N/A
